# Pr^3+^-doped YPO_4_ nanocrystal embedded into an optical fiber

**DOI:** 10.1038/s41598-024-57307-4

**Published:** 2024-03-28

**Authors:** Dominik Dorosz, Marcin Kochanowicz, Rafael Valiente, Andrea Diego-Rucabado, Fernando Rodríguez, Nuria Siñeriz-Niembro, José I. Espeso, Magdalena Lesniak, Piotr Miluski, Sylvia Conzendorf, Juliane Posseckardt, Zhongquan Liao, Gloria Lesly Jimenez, Robert Müller, Martin Lorenz, Anka Schwuchow, Martin Leich, Adrian Lorenz, Katrin Wondraczek, Matthias Jäger

**Affiliations:** 1 AGH University of Krakow, A. Mickiewicza Av. 30, 30-059 Kraków, Poland; 2 Bialystok University of Technology, Wiejska 45D Street, 15-351 Białystok, Poland; 3 University of Cantabria, Avenida. de Los Castros 48., 39005 Santander, Spain; 4 Fraunhofer Institute for Ceramic Technologies and Systems IKTS, Maria-Reiche-Str. 2, 01109 Dresden, Germany; 5 Leibniz Institute of Photonic Technology, Albert-Einstein-Str. 9, 07745 Jena, Germany

**Keywords:** Glass powder—NCs doping method, YPO_4_:Pr^3+^ nanoparticles, Nanocomposite optical fibers, Electronics, photonics and device physics, Materials for devices, Materials for optics, Nanoscale materials, Structural materials, Optical materials and structures, Other photonics

## Abstract

Optical fiber with YPO_4_:Pr^3+^ nanocrystals (NCs) is presented for the first time using the glass powder—NCs doping method. The method’s advantage is separate preparation of NCs and glass to preserve luminescent and optical properties of NCs once they are incorporated into optical fiber. The YPO_4_:Pr^3+^ nanocrystals were synthesized by the co-precipitation and hydrothermal methods, optimized for size (< 100 nm), shape, Pr^3+^ ions concentration (0.2 mol%), and emission lifetime. The core glass was selected from the non-silica P_2_O_5_-containing system with refractive index (*n* = 1.788) close to the NCs (*n*_o_ = 1.657, *n*_e_ = 1.838). Optical fiber was drawn by modified powder-in-tube method after pre-sintering of glass powder—YPO_4_:Pr^3+^ (wt 3%) mixture to form optical fiber preform. Luminescent properties of YPO_4_:Pr^3+^ and optical fiber showed their excellent agreement, including sharp Pr^3+^ emission at 600 nm (^1^D_2_–^3^H_4_) and ^1^D_2_ level lifetime (τ = 156 ± 5 µs) under 488 nm excitation. The distribution of the YPO_4_:Pr^3+^ NCs in optical fiber were analyzed by TEM-EDS in the core region (FIB-SEM-prepared). The successful usage of glass powder—NCs doping method was discussed in the aspect of promising properties of the first YPO_4_:Pr^3+^ doped optical fiber as a new way to develop active materials for lasing applications, among others.

## Introduction

Optical fibers doped with rare-earth ions (RE) have enabled incredible progress in developing lasers, optical amplifiers, and broadband sources. All due to the optical fiber’s unique construction, which provides a large volume of active core material, allowing sufficient gain for lasing or amplification. Optical fibers doped with RE ions are known for their few broadly applicable laser transitions in silica, fluoride, and chalcogenide glass matrix^1,2^. Considering the new laser transitions, they suffer certain limitations due to the high phonon energy and low acceptability of RE ions, in the case of the silica, and the low mechanical resistance for the fluoride and chalcogenide optical fibers. Since we do not have an ideal matrix that meets the possibilities of laser generation at the novel wavelengths in the broad UV-MIR range, new solutions are being sought by introducing RE-doped nanocrystals into the fiber core to meet laser requirements. This approach’s potential advantage is obtaining optical fiber sources with efficient emission by exploiting the properties of the active crystals, i.e., an ordered lanthanide environment with controlled phonon energy and RE concentration.

Currently, there are three main ways to obtain a fiber with a crystalline phase by: (i) controlled crystallisation, i.e. annealing of the fiber after fabrication^3^, (ii) introducing crystals into the glass prior to the fiber fabrication by different methods^4–7^ and (iii) NCs crystallisation during the process of fiber drawing^8,9^. Several fiber constructions with oxide and fluoride nano- and micro-crystals doped with Nd, Er, and Tm ions have been presented by controlled crystallisation, where an essential aspect was to determine the RE ion environment confirmed by structural studies leading to the increase of luminescence properties (e.g., intensity and lifetime). The method of direct introduction of RE-doped crystals into the glass suffers from micron-size crystal distribution and dissolution effect^5^. This is the result of the chemical interaction of the glass components on the crystal and the thermodynamic conditions of the solution at a specific temperature, strongly influencing the tendency towards dissolution, crystallisation or even recrystallisation. The influence of the glassy matrix composition was presented in a tellurite glass system with LiYF_4_:Er, Yb NCs, where an optical fiber was formed at the optimum temperature and dwelling time^4^. Another interesting approach benefits from the natural tendency to crystallisation, achieved in silica fiber, e.g., by doping silica soot with P_2_O_5_ and YAG crystals, where the crystallisation of the YPO_4_ phase was achieved after the prior dissolution of the YAG crystals. Additionally, a significant approach in controlling crystal size and their unique distribution relative to the fiber drawing temperature was pointed out, which determines Rayleigh light scattering in the fiber^10^. Despite the progress in developing fibers with active NCs, all techniques have the lack of laser action so far in common, mainly because of active crystal dissolution or aggregation and the low optical quality of the fiber. Undoubtedly, a key aspect is the NC resistance to dissolution, which is mainly the result of the interaction of the glass components on the crystal structure, leading to its gradual disintegration^11^.

A way to reduce the solubility of NCs can be the new approach proposed in this article to use the natural crystallisation tendency of a particular crystal as a mechanism for preventing its dissolution. We have observed such a phenomenon for YPO_4_ NCs doped with Pr^3+^ ions. This observation was preceded by introducing Y_2_O_3_:Pr^3+^ NCs into glasses containing P_2_O_5_ in their composition, in which, as in Ref.^10^, the crystallisation of the new YPO_4_:Pr^3+^ phase appeared. Interestingly, luminescent measurements confirmed that most of the Pr^3+^ ions are present in the YPO_4_ NCs, while a tiny fraction corresponds to Pr^3+^ in the glassy matrix. As a result, we recently pointed out the initial conditions for the fabrication of the optical fiber with YPO_4_:Pr^3+^^12^.

In this work, we demonstrate ex-situ glass powder doping with YPO_4_:Pr^3+^ NCs, which led to their survival in an optical fiber due to a novel concept of using a phosphate-containing glass providing a low tendency to crystallise of the YPO_4_ phase, preventing dissolution of initially YPO_4_:Pr^3+^ embedded NCs. The step-by-step manner to achieve this goal will be presented, showing at first the high tendency to crystallisation of the YPO_4_:Pr^3+^ phase before the dissolution of Y_2_O_3_:Pr^3+^ NCs in the phosphate-containing glass. This is followed by the synthesis and characterisation of YPO_4_:Pr^3+^ NCs and the choice of the host glass system. The glass powder NCs doping technique led to fabricating an optical fiber preform with YPO_4_:Pr^3+^ NCs embedded. In summary, optical fiber with a core containing YPO_4_:Pr^3+^ NCs was drawn, confirming their presence by TEM, FIB-SEM, and luminescence spectroscopy, including lifetime.

## Results and discussion

### NCs synthesis and characterization

Initially, we planned to use Y_2_O_3_:Pr^3+^ crystals that were introduced into various glasses, including glasses containing P_2_O_5_. However, there was usually a rapid dissolution of the crystalline phase or multi-phase crystallisation of the host glass. This was different in the case of the phosphate glasses, where crystallisation of a single YPO_4_:Pr^3+^ phase occurred. This drove us to analyse the potential increased insolubility of the YPO_4_ phase in the phosphate-containing glasses.

#### Y_2_O_3_:Pr^3+^ in a phosphate glass matrix

**Figure 1:**
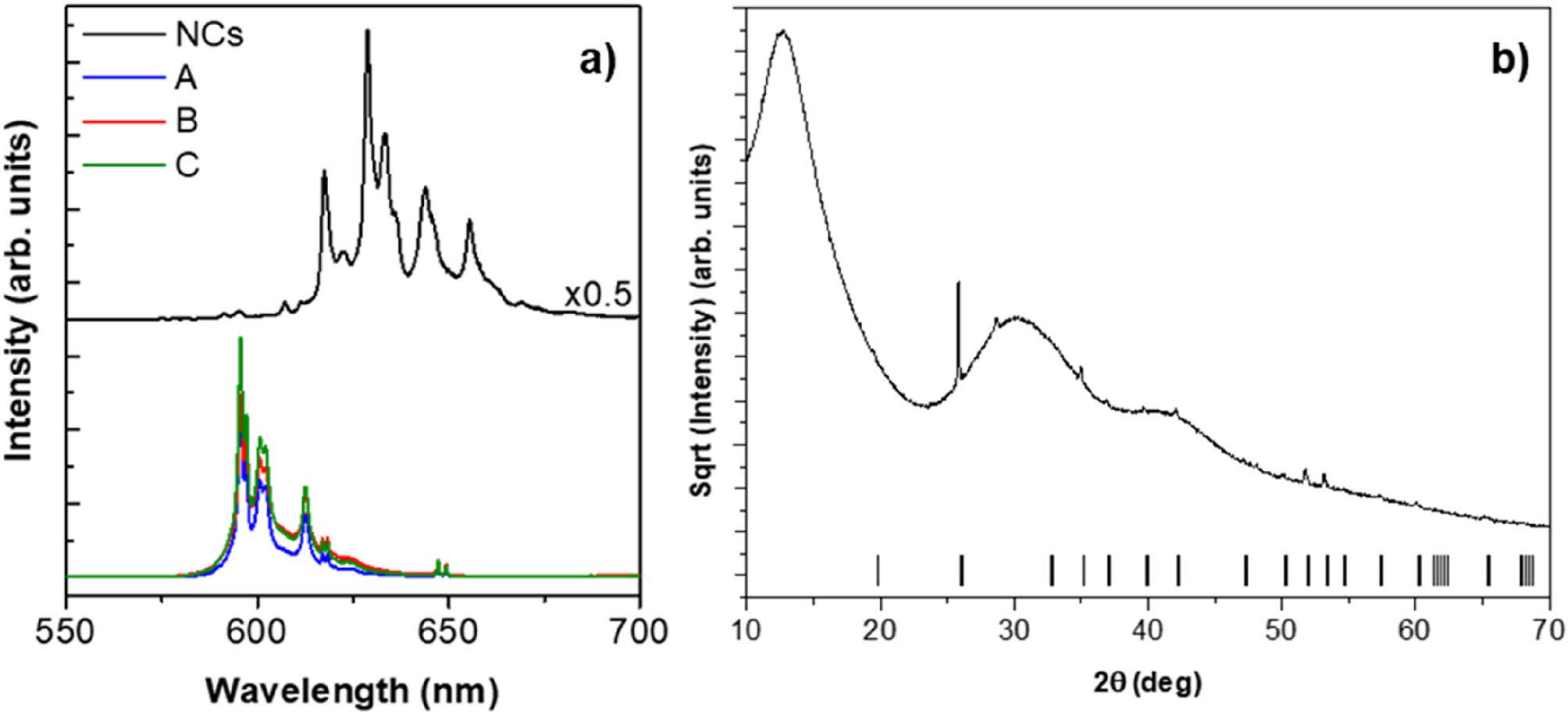
(**a**) RT emission spectra of the original Y_2_O_3_:Pr^3+^ NCs and the resulting NC-doped glass matrix at different spots (A, B, C); (**b**) powder XRD pattern of the resulting combination of glass and Y_2_O_3_:Pr^3+^ NCs. Vertical lines correspond to Bragg reflections from the YPO_4_ tetragonal phase.

Y_2_O_3_:Pr^3+^ NCs prepared through solvothermal method as described elsewhere^13^ were homogeneously mixed with glass powders and melted in an induction fusion fluxer equipment (Equilab F1) using a platinum crucible to study the stability and compatibility between both components. Emission spectra were recorded to check the survival of the NCs after melting. Interestingly, remarkable differences were detected from the resulting NC-doped glass while carrying out the spectroscopic characterization between the original Y_2_O_3_:Pr^3+^ NCs and the melted glass containing the NCs at different spots (A, B, C) (Fig. 1a). Sharp emission lines in the 580–630 nm range were detected, thus confirming the absence of Pr^3+^ in a glassy environment. However, the peak position, relative intensities between peaks, and the general shape of the emission spectrum remarkably differed from that of the original Y_2_O_3_:Pr^3+^ NCs. Indeed, no remaining emission peaks of Pr^3+^-doped Y_2_O_3_ could be detected. Therefore a complete transformation of the original NCs to an entirely different crystalline phase was expected. Furthermore, the sharp features do not match what is expected for Pr^3+^ in a glass matrix.

XRD measurements were performed to check the newly formed phase. After pattern refinement of the sharp, narrow diffraction peaks over the broad glassy background, these peaks were assigned to the YPO_4_ tetragonal phase (JCPDS 011-0254) (Fig. 1b). A search in the archive literature confirmed that the resulting emission spectrum perfectly matched the Pr^3+^-doped YPO_4_ emission spectrum reported by Collins et al.^14^.

According to the stability demonstrated by this new Pr^3+^-doped phosphate phase and its sharp and intense luminescence within the glass environment, introducing YPO_4_ NCs directly into the glass powders was proposed. For such purpose, the synthesis of YPO_4_:Pr^3+^ NCs was carried out, and after their structural and optical characterization, the survival of such NCs during the fiber drawing process was studied.

#### YPO_4_:Pr^3+^ NCs

**Figure 2:**
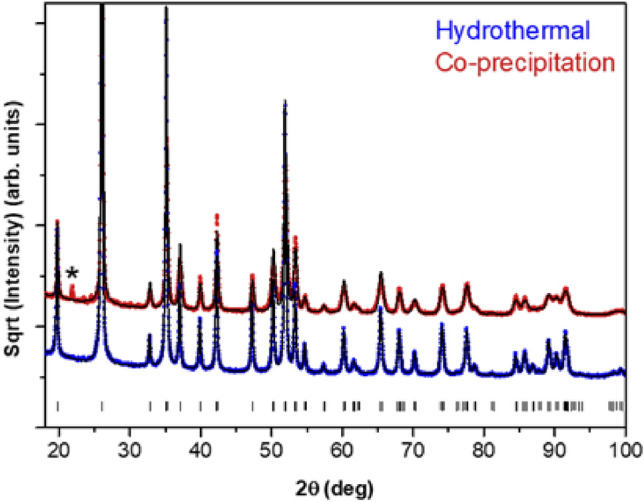
XRD pattern of the YPO_4_:Pr^3+^ (0.2%) NCs prepared via co-precipitation synthesis. Vertical lines correspond to Bragg reflections from the tetragonal YPO_4_ phase. *Indicates the hydrated hexagonal YPO_4_ phase.

XRD patterns from YPO_4_:Pr^3+^ (0.2%) NCs obtained by the co-precipitation and hydrothermal methods are displayed in Fig. 2. Both procedures yield a tetragonal YPO_4_ phase (space group *I4*_*1*_*/amd*), also referred as xenotime. A small peak at around 21° was also detected for NCs prepared through the co-precipitation method, which is characteristic of the Y-phosphate hexagonal hydrated phase (YPO_4_·0.08H_2_O), also known as churchite (space group P_6_222), because of the lower calcination temperature applied in such procedure. Even though the transformation temperature from hexagonal (hydrated) to tetragonal (dehydrated) phase has been reported to be *ca*. 915 °C, it appears that higher temperatures or longer calcination times were necessary during the thermal treatment to achieve the complete transformation to xenotime phase^15^. In this sense, the remaining hydrated phase from co-precipitation NCs, which is absent in the hydrothermal synthesis method, is expected to be removed entirely during the fiber drawing process since higher temperatures are required. No other impurity traces were detected within the experimental uncertainty, and an average crystallite size of 32 nm and 60 nm were estimated by Rietveld refinement for NCs prepared by co-precipitation and hydrothermal methods, respectively.

**Figure 3:**
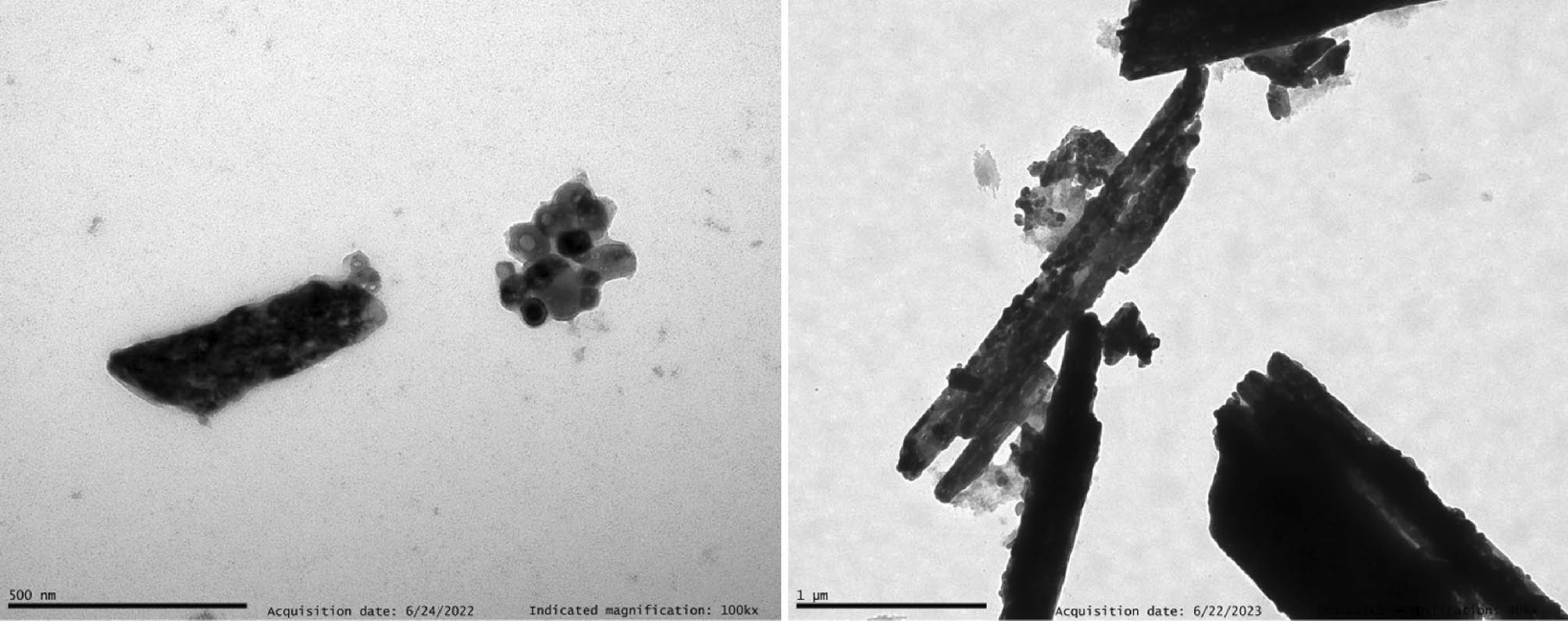
TEM image of the YPO_4_:Pr^3+^ (0.2%) NCs prepared through co-precipitation synthesis method.

**Figure 4:**
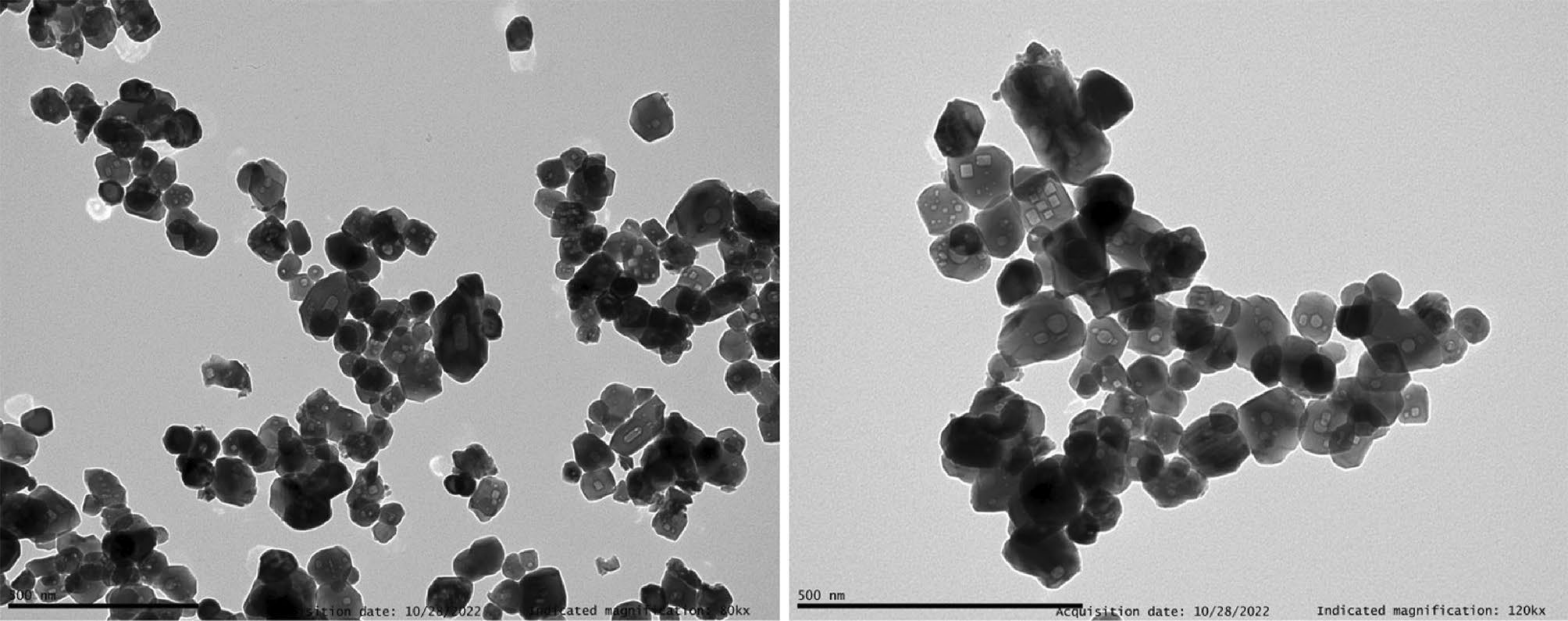
TEM image of the YPO_4_:Pr^3+^ (0.2%) NCs prepared through hydrothermal synthesis method.

On the other hand, different morphologies and NC sizes were obtained as a function of the synthesis procedure. Firstly, a high degree of inhomogeneity in shape and particle size could be detected from TEM images for NCs prepared through the co-precipitation method, as shown in Fig. 3. A mixture of micrometric rod-like particles and small rounded NCs was observed. Specifically, the former present diameters ranging from 250 to 300 nm and lengths in the micron range, while the NCs present a 25–60 nm size range. Interestingly, as it can be observed from Fig. 3, it seems that these big rod-like particles are formed by the aggregation of NCs, which would be in good agreement with the average NC size estimated from the Rietveld refinement. On the other hand, hydrothermal synthesis allowed to obtain well-defined NCs with an excellent grade of dispersion and crystallite sizes with a 60–100 nm size range, as displayed in Fig. 4, again in good agreement with results from Rietveld refinement.

**Figure 5:**
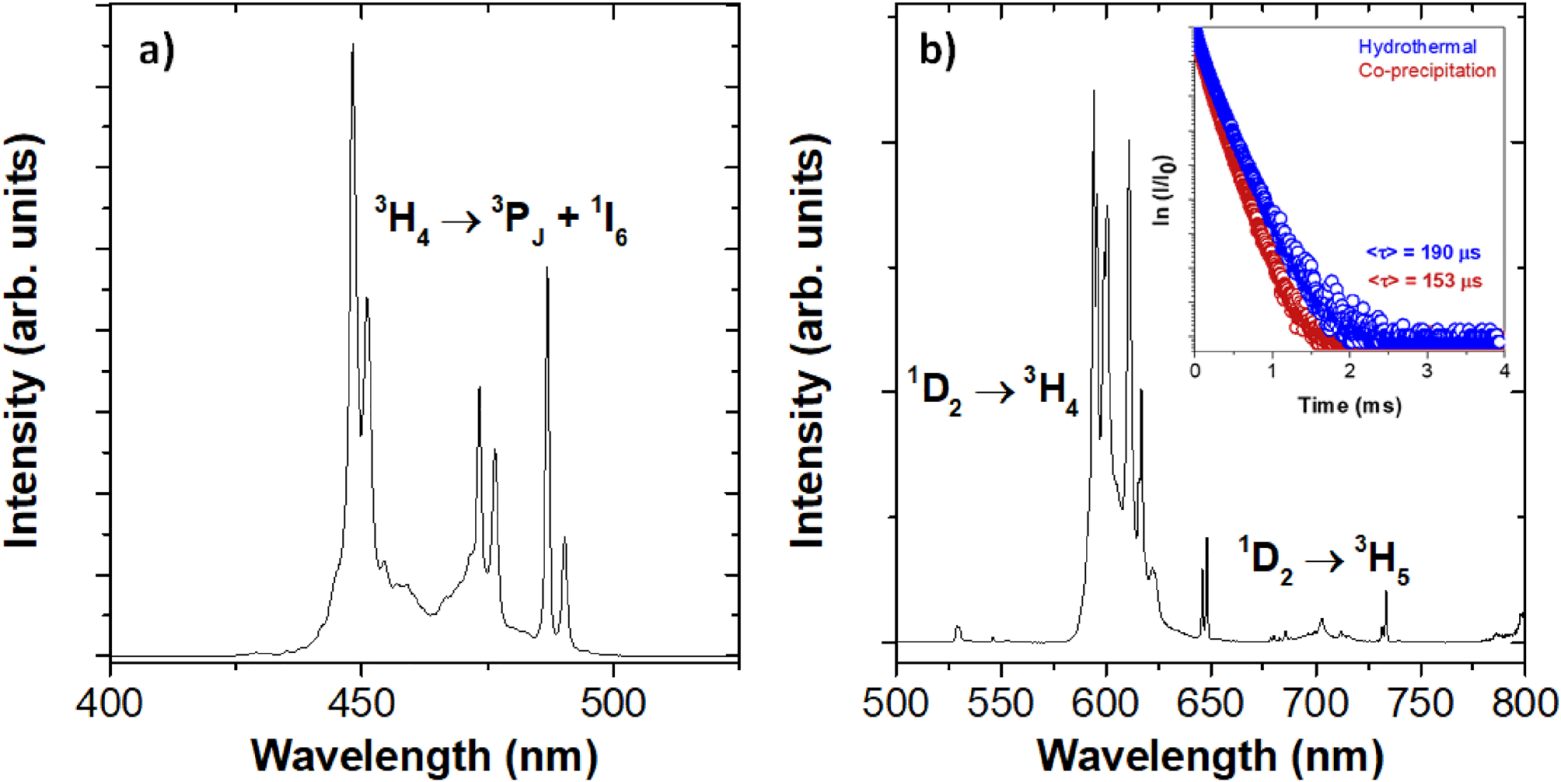
(**a**) RT excitation (λ_em_ = 595 nm) and (**b**) emission (λ_exc_ = 448 nm) spectra of YPO_4_:Pr^3+^ (0.2 mol%) NCs prepared through hydrothermal method and RT time evolution of the ^1^D_2_ to ^3^H_4_ emission of YPO_4_:Pr^3+^ (0.2 mol%) NCs synthesized via co-precipitation and hydrothermal methods (inset).

RT excitation and emission spectra of the YPO_4_:Pr^3+^ (0.2%) NCs prepared using the hydrothermal method are displayed in Fig. 5. The excitation spectrum was recorded after monitoring the emission from the ^1^D_2_ multiplet to the ^3^H_4_ ground state (λ_em_ = 595 nm) (Fig. 5a). The spectrum is dominated by sharp lines in the 430–500 nm spectral range, attributed to the intraconfigurational transitions from the ^3^H_4_ ground state to the ^3^P_J_ + ^1^I_6_ multiplets. On the other hand, the emission spectrum was obtained upon direct Pr^3+^ excitation at 448 nm into the ^3^P_J_ multiplet (Fig. 5b). The spectrum exhibits peaks located in the 580 to 630 nm region, which are characteristic of emission from ^1^D_2_ to ^3^H_4_ level, while peaks located in the range of 670–750 nm are assigned to the transition ^1^D_2_ → ^3^H_5_. Emission from the ^3^P_0_ level is very weak in YPO_4_: Pr^3+^^13^. Additionally, the time evolution of the luminescence intensity from the ^1^D_2_ excited state to the ^3^H_4_ ground state of Pr^3+^ in YPO_4_ for NCs prepared by both co-precipitation and hydrothermal methods was monitored. For this purpose, emission at 595 nm was recorded after excitation into the ^3^P_J_ multiplet at 448 nm (Fig. 5b inset). The emission decay curves were fitted to a double exponential function, obtaining an average lifetime of <τ>  = 153 μs for NCs prepared through the co-precipitation method and <τ>  = 190 μs for NCs obtained by the hydrothermal method for the same nominal concentration (0.2 mol%). Differences in average decay rates were attributed to the applied thermal treatments. Specifically, not only the higher temperature employed in hydrothermal synthesis (1200 °C) promoted a better crystallinity but also completely removed the YPO_4_ hydrated phase, as observed in Fig. 3. In this sense, both features reduced non-radiative relaxation processes and thus, resulted in a longer emission lifetime of Pr^3+^ when compared with the NCs prepared by co-precipitation synthesis.

### Optical fiber with YPO_4_:Pr^3+^ NCs

**Figure 6:**
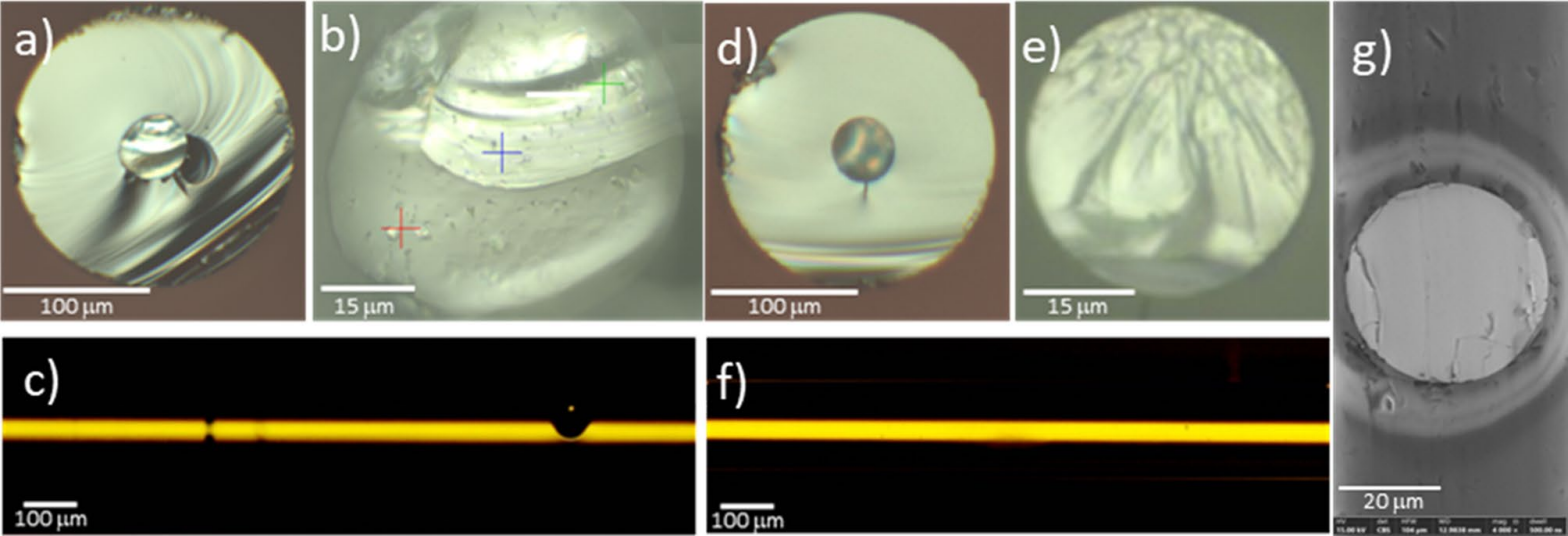
Optical fiber cross-sections (**a–c**) with bubbles and (**d–f**) without bubbles in the core region; (**c,f**) side views of the fibers; (**g**) SEM cross-section of the fiber’s core without bubbles.

The thermal conditions for the optical fiber drawing differed from the typical rod-in-tube method. This was due to a technique for preparing the core material involving isostatic forming of the glass-NCs mixture and then the pre-sintering step. These resulted in a densification level of 90% of the core material, but the rest of the volume representing gas was disposed of during core consolidation at the fiber drawing process. Optimisation of the consolidation time and temperature was crucial to avoid bubbles in the core volume (Fig. 6a, b). Consequently, a underpressure of 1000 Pa was applied during the drawing process, which produced longer fibers (~ 1 m) without bubbles in the core (Fig. 6d, e, g). The microscopic images of a side view of both fibers are presented as the insets in the Fig. 6c, f.

**Figure 7:**
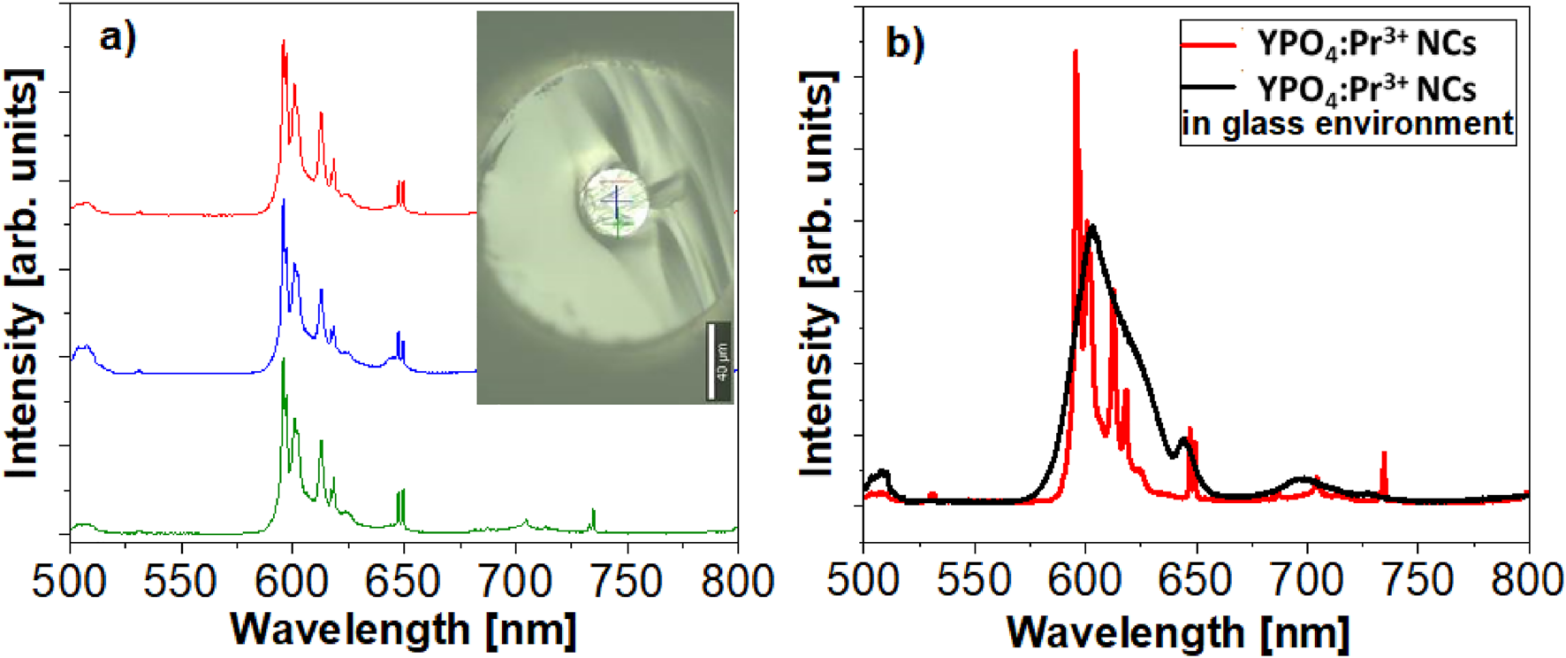
Luminescence (λ_exc_ = 448 nm) (**a**) mapping of optical fiber’s core with YPO_4_:Pr^3+^ NCs with marked measurement points (inset) and (**b**) spectra of the YPO_4_:Pr^3+^ NCs (red curve) and glass with dissolved NCs (black curve).

The presence of YPO_4_:Pr^3+^ NCs was verified by mapping the core luminescence under the 488 nm laser excitation with a spot diameter of 2 μm (Fig. 7a). The characteristics obtained are in excellent agreement with the luminescence spectrum of the as-grown NCs (Fig. 5b). In contrast, when the dissolution of NCs occurred, broad emission lines of the Pr^3+^ were visible, which is characteristic of amorphous environment (Fig. 7b—black curve).

**Figure 8:**
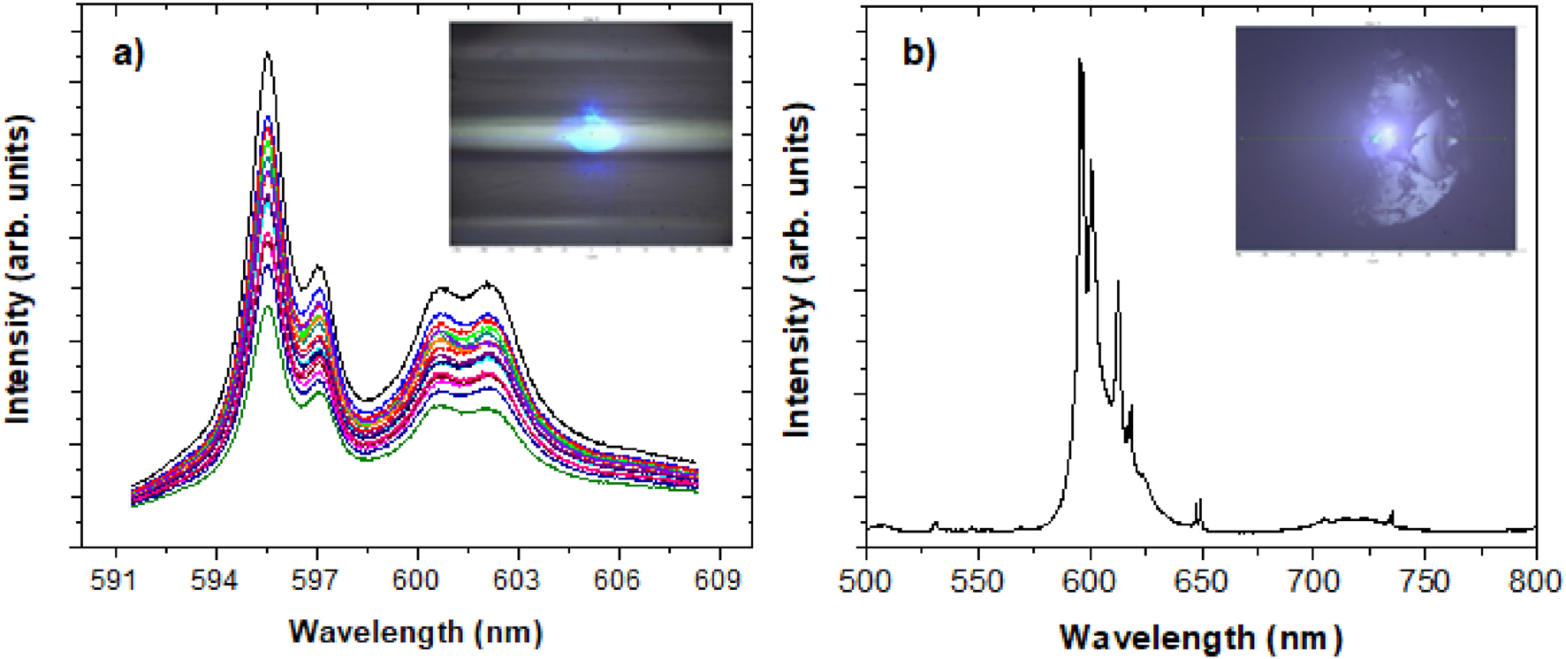
Emission spectra (λ_exc_ = 488 nm) (**a**) along the fiber focusing the laser line at different positions of the fiber (laser spot 5 μm, spectra were taken every 20 μm) and (**b**) from the fiber core (inset: laser spot focused on the fiber core).

Additionally, emission spectra of YPO_4_:Pr^3+^ (0.2%) NCs embedded in the glass fiber were measured at different distribution fiber points to demonstrate NCs distribution homogeneity along the fiber core. It was possible to obtain the Pr^3+^ luminescence spectrum by exciting with the laser beam transversely to the fiber (Fig. 8a). The observed emission spectrum is identical to the Pr^3+^-doped YPO_4_ (λ_exc_ = 488 nm), demonstrating that NCs survived the fiber drawing process (Fig. 8b).

**Figure 9:**
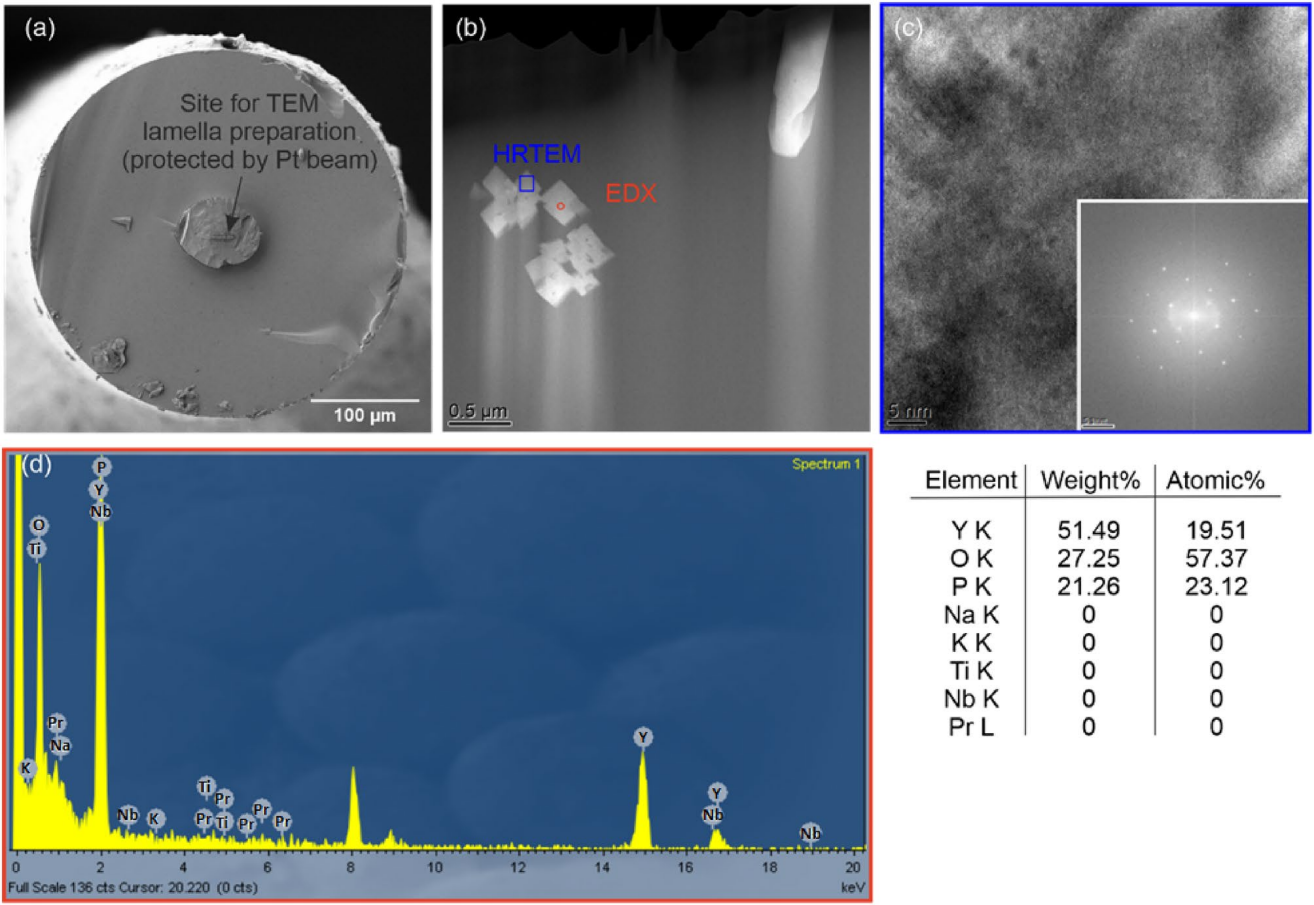
TEM analysis. (**a**) SEM image of the fiber indicating the site of preparation. (**b**) STEM dark field image of the lamella. (**c**) HRTEM image of the indicated NC. The inset shows FFT pattern. (**d**) EDX analysis of the NC. The presence of Cu originates from the TEM grid. Elements of the matrix were included into the analysis. Pr^3+^ was below the detection limit.

The luminescence spectrum does not provide information on the real microscopic distribution and size of NCs in the fiber core because the beam is focused with a spot of about 2 nm width. Both aspects are relevant for lasing action in optical fiber since they lead to lifetime reduction of the upper laser level and increasing scattering^10,16^. Therefore, TEM analysis of the FIB-SEM-prepared core region was performed. The structure of the observed NCs was confirmed by high-resolution TEM (HRTEM), including fast Fourier transform (FFT) patterns and their chemical composition by energy-dispersive X-ray (EDX) diffraction analysis (Fig. 9).

**Figure 10:**
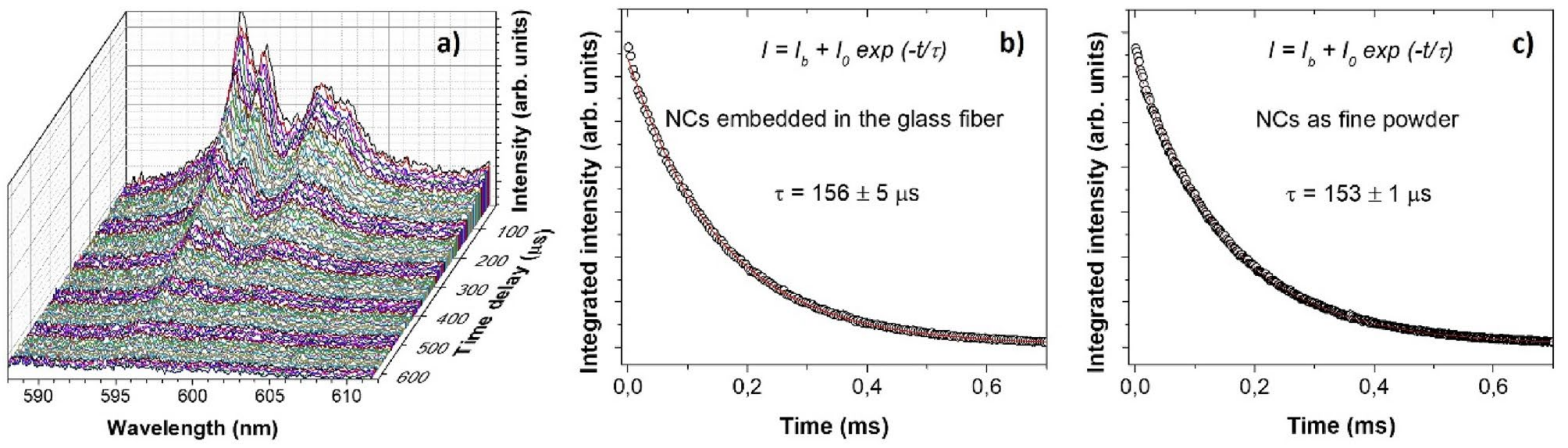
(**a**) Time-resolved emission spectrum of co-precipitation YPO_4_: Pr^3+^ (0.2 mol%) NCs embedded into the glass fiber. Excitation wavelength 488 nm using different delay times after modulated excitation (frequency, *f* = 333 Hz). (**b**) Time dependence of the integrated intensity of the spectra corresponding to different delay times. (**c**) Emission lifetime of YPO_4_ NCs (0.2 mol%) growth by the co-precipitation method.

The NCs located in the core show a certain tendency to localise in larger groups of NCs along the core axis. This effect is a known result from the glass mass’s centripetal viscous flow forces during the fiber drawing process^16^. However, it does not influence on luminescent properties of the NCs in the fiber, which is evidenced by the measurement of the lifetime. The temporal evolution of the photoluminescence spectra around the maxima of the emission peaks is plotted in Fig. 10a. The integrated intensity of these spectra at different delay times gave the emission lifetime of the ^1^D_2_ state (τ = 156 ± 5 µs, Fig. 10b), in excellent agreement with the value reported by the co-precipitation NCs as fine powders in the same conditions (τ = 153.5 ± 0.1 µs, Fig. 10c).

It was shown that YPO_4_:Pr^3+^ NCs are resistant to dissolution in the Nb_2_O_5_–P_2_O_5_–Na_2_O–TiO_2_–K_2_O glass system, which allowed them to survive the optical fiber drawing process. Analysing this result in relation to the current discussion about the NCs solubility in glasses, the two aspects are essential, the influence of the glass components and its viscosity during clarification of glass-NCs mixture. The first one is difficult to determine, as the multicomponent and non-silica glass were used. The chosen glass had to fulfill such basic requirements as resistance to crystallisation during drawing as well as being filled with NCs and matching the refractive index. Then, the optimisation of core preparation and optical fiber drawing led to the evaluation of NCs dissolution rate. In previous investigations, authors carried out several optical fiber drawing tests, including silicates, germanates, and tellurites glasses, indicating, in those cases, the full or almost complete dissolution of Y_2_O_3_ and YPO_4_ NCs. In this sense, we claim that analyzing glass composition vs. dissolution is rather a difficult task. One important factor is the influence of glass viscosity on NCs solubility. It was also confirmed recently in Ref.^10^, where increased solubility of YPO_4_ NCs was discovered in silica glass during fiber drawing in 2000 °C. It is also important to note that the dissolution of YPO_4_ increased after the introduction of P_2_O_5_ which lowered the viscosity of the silicate glass. It is obviously impossible to compare those two glass systems as they are opposed in terms of glass composition. Still, the increase in ion mobility undoubtedly affects the dissolution rate of the NCs. It was also noticed during our optical fiber drawing studies performed for the same temperature, i.e., 1150 °C, using glasses with different compositions (silicates, germanates) but similar viscosity (~ 10^–3^–10^–4^ dPas), finding that survival of YPO_4_ NCs occurred only at higher viscosity. The issue here is that the majority viscosity required for core clarification (Fig. 7a) is too low to prevent NCs dissolution and only in P_2_O_5_-containing glass system was achieved. A possible explanation of the role of the crystallisation tendency of the YPO_4_:Pr^3+^ phase in the presence of P_2_O_5_ can be identified. This tendency was demonstrated in an experiment where the introduction of Y_2_O_3_ NCs was followed by melting and crystallisation as YPO_4_ (Fig. 2). When YPO_4_:Pr^3+^ NCs are embedded into the glass, the tendency to crystallise occurs when they are partially dissolved, and Y^3+^ cations appear. Hence, this is believed to slow down the solubility of the NCs in the selected glass with P_2_O_5_, while for other compositions, a dissolution of the YPO_4_:Pr^3+^ phase occurs. In optical fiber drawing at higher temperatures, above 1200 °C, dissolution of the NCs occurs (Fig. 7b), but no recrystallisation due to the fast cooling of the fiber leaving the hot zone of the furnace. Therefore, the recrystallisation of YPO_4_ cannot also occurs at the lower temperature of 1150 °C—which was used for fiber drawing, i.e., with survived YPO_4_:Pr^3+^ NCs in the core. This is confirmed by the results of the lifetimes of the NCs in the optical fiber, which are consistent with the synthesised NCs (Fig. 10). Even though there is difficulty in pointing out the clear mechanism preventing YPO_4_:Pr^3+^ NCs dissolution, we considered that the content of P_2_O_5_ (21 wt%) may play a supporting role. Our preliminary studies showed that low concentration of P_2_O_5_ (< 10 wt%) tends to the fast NCs dissolution and even multi-phase crystallization. On the other hand, high concentration of P_2_O_5_ (> 60 wt%) promotes full NCs dissolution without crystallization of any phase. It seems to be the range of P_2_O_5_ content (~ 5–10 wt%—depends on the glass composition) for which the PO_x_ units are not playing a glass-forming role. Therefore, the tendency to form PO_x_ unit is weak and thus does not clearly affect the dissolution of YPO_4_ crystals since P_2_O_5_ is not a main former oxide in the glass.

Furthermore, in the TEM image, a tendency for the orientation of the NCs along the fiber can be seen, which relates to a lateral compressive force appearing during optical fiber drawing. It has been reported that NCs tend to present a preferential orientation of their c-axis along the rod within the fiber during the drawing process^6^. The conclusive argument would be the EDX analysis of the core region for the presence of Pr^3+^ ions. Unfortunately, the elemental composition of the NCs in the fibers regarding the presence of Pr^3+^ ions was not clearly established due to their low content of 0.2 mol%, which is beyond the TEM–EDX probe detection. Such studies will soon be performed on optical fibers with higher lanthanide content in NCs.

## Conclusions

The growing interest in nanocomposite NCs doped optical fibers is connected with the demand for new optical fiber laser transitions available in RE-doped NCs. The main issue is to preserve their superior luminescent properties in the optical fiber with regard to the fiber losses resulting from the nanoparticle size, spatial distribution, and refractive index matching of the NCs with the glass matrix. We proposed to fulfill those requirements by using the glass powder—NCs doping method, showing nanocomposite optical fiber doped with YPO_4_:Pr^3+^ NCs for the first time. The advantage of this approach over the other methods is the separate preparation of NCs and glass matrix, which allowed to optimise a refractive index matching (glass-NCs) and preserve luminescent properties (λ = 600 nm, ^1^D_2_–^3^H_4_, lifetime τ = 156 ± 5 µs) of YPO_4_:Pr^3+^ NCs embedded in the optical fiber. The key step relied on using a modified powder-in-tube method and selection of non-silica P_2_O_5_ containing glass matrix, which allowed drawing of optical fiber after prior pre-sintering of optical fiber preform.

The survival of the YPO_4_:Pr^3+^ in optical fiber were confirmed by luminescent and lifetime measurements of NCs, showing an excellent agreement with the properties of NCs as fine powder. The TEM–EDX analysis of the FIB-SEM-prepared lamella of optical fiber core region showed the presence of the NCs.

The discussion about the successful usage of glass powder—NCs doping method directed to consider the tendency to crystallisation of NCs in the chosen glass system as a supporting mechanism decreasing the dissolution of NCs during the optical fiber drawing process. The first nanocomposite YPO_4_:Pr^3+^ doped optical fiber can be a new way to develop active materials for lasing applications.

## Methods

### NCs synthesis and characterization

#### YPO_4_:Pr^3+^ NCs

YPO_4_:Pr^3+^ NCs were prepared following a modified co-precipitation method described elsewhere^17,18^. Y(NO_3_)_3_ and the corresponding stoichiometric amount of Pr(NO_3_)_3_ for a 0.2 mol% Pr^3+^ concentration were dissolved in deionized water. Then, twice the stoichiometric amount of NH_4_H_2_PO_4_ was added, and the mixture was stirred at room temperature (RT) for 20 min. The precipitated solid was separated by centrifugation, washed three times with deionized water, and suspended in ethanol to be dried over night (o.n.) at 80 °C. A final thermal treatment at 950 °C for 2 h with a heating ramp of 15 °C/min was applied to avoid the formation of the hydrated phase.

A modified protocol of the procedure described by Zou et al.^19^ was also followed to synthesize YPO_4_:Pr^3+^ (0.2%) NCs. Specifically, Y(NO_3_)_3_ and the corresponding stoichiometric amount of Pr(NO_3_)_3_ were dissolved in deionized water. Then, twice the stoichiometric amount of NH_4_H_2_PO_4_ was added, and the mixture was stirred at RT for 5 min. The mixture was transferred to a Teflon-lined stainless-steel autoclave and heated for 24 h at 160 °C. After naturally cooling down to RT, the solid was separated by means of centrifugation and washed twice with deionized water and once with ethanol. After drying o.n. at 80 °C, powders were calcinated at 1200 °C for 2 h with a heating ramp of 15 °C/min.

#### Structural and optical characterization of the NCs

X-ray diffraction (XRD) patterns were acquired with a Bruker D8 Advanced diffractometer equipped with a Cu tube (wavelength: <Kα1,2>  = 1.5418 Å) and a fast LYNXEYE 1D-detector. NCs diffraction pattern were measured in the 10°–100° range (2θ) for phase identification, quantification, and structure refinement. Rietveld refinement was carried out to estimate the average crystallite size of the NCs as well as the lattice parameters.

Transmission electron microscopy (TEM) images were obtained to study both the morphology and particle size of the NCs using a JEOL JEM-1011 electron microscope equipped with a high-resolution CCD camera (Gatan).

RT optical spectroscopy of the NCs was studied, recording the excitation and emission spectra with a FLS920 spectrofluorometer from Edinburgh Inst. equipped with double monochromators in emission and excitation, a 450 W Xe lamp as a CW excitation source and an electrically cooled photomultiplier tube R928P (Hamamatsu) for detection. Emission lifetime measurements were performed with a 60 W pulsed Xe lamp.

### Optical fiber development and characterization

#### Optical fiber drawing method

The widely known rod-in-tube method was used to fabricate an optical fiber. Optical fiber preform was prepared in a separated stage, where the core material was composed of a densified mixture of glass (Ohara S-NPH7) powder from the 54Nb_2_O_5_–21P_2_O_5_–15Na_2_O–6TiO_2_–4K_2_O (wt%) system (refractive index *n* = 1.78 @ λ = 633 nm) and 3 wt% of YPO_4_:Pr^3+^ NCs (co-precipitation). The glass was ground using an E-max planetary high-energy mill (Retsch GmbH, Germany) using a 650–1200 rpm speed to obtain particles with a size distribution of 500–900 nm. A slurry of the mixture in ethanol was homogenised using an ultrasonic sonotrode mixer (Sonics Vibra cell, 500 W, 20 kHz, 5 min), dried at 200 °C and then prepared as rods moulded in an isostatic press at 250 MPa. Subsequently, sintering was carried out at 650 °C, obtaining a preform core with a diameter of 4 mm. To confirm the homogeneous distribution of the NCs within the mixture before and after sintering, SEM/EDX measurements (company) and Raman mapping (Witec Alpha 300 M+) were performed under 488 nm laser excitation. The prepared core material was placed in a 10 mm/5 mm (outer/inner diameter, respectively) DURAN glass tube (Schott), used as cladding. The optical fibers were extracted at a speed of 25 m/min at 1150 °C, corresponding to a viscosity of the cladding glass of 10^4.5^ dPas, using a fiber drawing tower with a low-temperature multicomponent glass furnace.

#### Optical fiber characterization

The quality of the optical fibers was initially analysed using an optical microscope to determine the core’s continuity and the bubbles’ presence. Subsequently, the core cross-section was analysed using micro-Raman stand for mapping with a 488 nm laser diode through the characteristic red luminescence spectrum of YPO_4_:Pr NCs. For the chemical and nano-structural characterization, a TEM lamella was prepared by lift-out technique using a Dual-Beam SEM–FIB system (Carl Zeiss NVision40) and subsequent analysis thereof using a scanning TEM (Carl Zeiss Libra 200 MC Cs).

Additionally, the emission lifetime of the as-obtained YPO_4_:Pr^3+^ NCs was compared with those embedded in the fiber. For this purpose, the 488 nm laser line of an Ar^+^–Kr^+^ laser (Coherent Innova Spectrum 70C) was modulated with a chopper (f = 333 Hz), and the emission was detected with an intensified CCD camera (Jobin–Yvon iCCD3553) attached to a Triax-320 monochromator. The emitted light was detected using different delay times and gate times.

## Data Availability

The datasets used and/or analysed during the current study available from the corresponding author on reasonable request.
